# The effects of a low-carbohydrate, ketogenic diet on the polycystic ovary syndrome: A pilot study

**DOI:** 10.1186/1743-7075-2-35

**Published:** 2005-12-16

**Authors:** John C Mavropoulos, William S Yancy, Juanita Hepburn, Eric C Westman

**Affiliations:** 1 Division of General Internal Medicine, Department of Medicine, Duke University Medical Center, Durham, North Carolina, USA; 2 Center for Health Services Research in Primary Care, Durham Veterans Affairs Medical Center, Durham, North Carolina, USA

## Abstract

**Background:**

Polycystic ovary syndrome (PCOS) is the most common endocrine disorder affecting women of reproductive age and is associated with obesity, hyperinsulinemia, and insulin resistance. Because low carbohydrate diets have been shown to reduce insulin resistance, this pilot study investigated the six-month metabolic and endocrine effects of a low-carbohydrate, ketogenic diet (LCKD) on overweight and obese women with PCOS.

**Results:**

Eleven women with a body mass index >27 kg/m^2 ^and a clinical diagnosis of PCOS were recruited from the community. They were instructed to limit their carbohydrate intake to 20 grams or less per day for 24 weeks. Participants returned every two weeks to an outpatient research clinic for measurements and reinforcement of dietary instruction. In the 5 women who completed the study, there were significant reductions from baseline to 24 weeks in body weight (-12%), percent free testosterone (-22%), LH/FSH ratio (-36%), and fasting insulin (-54%). There were non-significant decreases in insulin, glucose, testosterone, HgbA1c, triglyceride, and perceived body hair. Two women became pregnant despite previous infertility problems.

**Conclusion:**

In this pilot study, a LCKD led to significant improvement in weight, percent free testosterone, LH/FSH ratio, and fasting insulin in women with obesity and PCOS over a 24 week period.

## Background

Polycystic ovary syndrome (PCOS) is the most common endocrine disorder among women of reproductive age, affecting approximately 4% of women [1]. PCOS is often associated with symptoms of excess testosterone: irregular or absent menses, excessive body hair, and infertility. PCOS is also associated with medical abnormalities such as central obesity [2], insulin resistance [3], hyperinsulinemia [4], type 2 diabetes mellitus [5], and dyslipidemia [6].

There are no known curative therapies for PCOS, though anti-diabetic medications do improve many of the metabolic abnormalities, like insulin resistance [7-11], and elevated serum testosterone and total cholesterol levels.[12,13] Dietary and exercise interventions [14,15] also have some impact on improving insulin sensitivity. In general, therapies that lower insulin levels and insulin resistance and lead to weight loss may prove useful for treating PCOS.

Recent studies have shown that a low-carbohydrate, ketogenic diet can lead to weight loss and improvements in insulin resistance [16,17]. Because weight loss and improving insulin resistance may be beneficial for PCOS, we performed this pilot study using a LCKD in women with PCOS.

## Methods

### Subjects

Subjects were recruited from the Raleigh/Durham/Chapel Hill areas in North Carolina through a community PCOS support group and by word of mouth. After meeting initial eligibility criteria by phone, including replying "yes" to the question, "Have you been told by your health care provider that you have PCOS?," subjects were asked to attend a screening visit for a medical history and physical exam. Informed consent approved by the local Institutional Review Board was obtained. Baseline blood tests were also performed at the screening visit. There were no monetary incentives for participation.

### Inclusion/exclusion criteria

The inclusion criteria were age 18–45 years, diagnosis suggestive of PCOS based on history of chronic anovulation and/or hyperandrogenemia, no other serious medical condition requiring medical supervision, body mass index (BMI) greater than or equal to 27 kg/m^2^, willingness to use acceptable contraception, and a desire to lose weight. Exclusion criteria included pregnancy, nursing or positive pregnancy test during screening period, and rapid progression of hyperandrogenic signs and symptoms.

### Intervention

Subjects received an intensive group education program during monthly group meetings held every other week throughout the 6-month study period. During the first group meeting, subjects were instructed on both the rationale and implementation of the dietary intervention via use of a LCKD diet book and handouts containing suggestions on choice of appropriate foods.[18] Subjects were then instructed to begin the diet the following day. During follow-up group meetings, study outcome measures were obtained, and continued dietary counseling, adjustment of individual medications, supportive counseling, sharing of food choices, and review of urinary ketones were performed. The duration of each meeting was approximately 1 hour.

Subjects were instructed to follow the LCKD, consisting of fewer than 20 grams of carbohydrate per day, as tolerated throughout the 6-month study period. The diet includes unlimited consumption of animal foods (meat, chicken, turkey, other fowl, fish, shellfish), prepared and fresh cheeses (up to 4 and 2 ounces per day, respectively), unlimited eggs, salad vegetables (2 cupfuls per day), and low carbohydrate vegetables (1 cupful per day). Subjects were strongly encouraged to drink at least six 8-ounce glasses of permitted fluids per day, and discouraged to drink caffeine and alcohol. Subjects were also encouraged to take one multivitamin per day and to exercise at least three times per week on their own, although this was not mandatory.

### Outcome measures

At the screening visit, baseline variables included age, gender, race, height, weight, prior use of weight loss programs, blood pressure, and laboratory tests. During the study, dietary adherence was measured by food records, self-report, and urinary ketones. Five-day food records for the days immediately preceding an upcoming group meeting were collected at baseline and weeks 2, 4, 12, and 24. Most dieters not on an LCKD do not have urinary ketones. As the intake of fewer than 20 g/day of carbohydrate typically results in urinary excretion of ketones, the presence of ketonuria was used to verify dietary adherence. (Urinary ketones were measured on a scale of 0="none" to 5="Large 160.")

Body weight was measured at each visit on the same scale with the subject wearing light clothing but with shoes and socks removed. (Tanita Model TBF-300A, Tanita Corp., Arlington Heights, Illinois) At all return visits, blood pressure was measured in the nondominant arm, using an automated digital cuff after sitting for 3 minutes (Omron Model HEM-725C, Omron Corp., Vernon Hills, Illinois). Two measurements were taken at each visit and averaged for the analysis. Blood tests were taken at baseline, 10, and 24 weeks after a 12 hour fast. Serum total and free testosterone were measured by immunoassay and equilibrium ultrafiltration; insulin by chemiluminescent immunometric assay.

A self-administered PCOS-specific questionnaire was completed by each subject during baseline and during each follow-up visit in order to monitor for changes in subjective symptoms related to PCOS.[19,20] The PCOS-Q includes 25 items from five health related quality of life domains: emotions (7 items), hair growth (5 items), body weight (5 items), infertility (5 items), and menstruation (4 items). Each item is rated on a seven-point scale in which a score of 7 indicates no problems or difficulties and a 1 indicates maximum impairment on that item. The mean score of all items in a domain provides a domain score for each subject.

### Statistical analyses

Because this pilot study used a "pre-post" design and the comparison of interest was the percent change from baseline to 24 weeks, a two-tailed paired *t *test was used to test for statistical significance of outcome variables. A p value of ≤ 0.05 was used for statistical significance.

## Results

Twenty-five women were screened by telephone; 12 remained eligible after screening and were invited to a screening visit. Eleven women retained eligibility after the screening visit and were enrolled in the study. Six subjects (54%) attended visits through 8 weeks, whereas 5 (45%) attended visits through 24 weeks. No subject dropped out due to reported symptomatic adverse effects. Two subjects were unable to comply with the diet program due to food preferences, two failed to follow the appointment schedule, and two were lost to follow-up. The mean age of subjects was 34.5 years, 80% were Caucasian, the mean weight was 102.5 kg, and the mean body mass index was 38.5 kg/m2.

### Program adherence

All five subjects developed ketonuria. The mean level of ketonuria for the entire study was 2.8 ("trace" to "small"), p < 0.0001. Inspection of five-day food records collected at weeks 2, 4, 12, and 24 indicated dietary compliance.

### Body weight

All subjects who participated through 24 weeks lost weight. The overall mean body weight change from baseline to 24 weeks was -12.1% (range: -4.0% to 16.4%) representing a mean decrease in BMI of 4.0 kg/m^2 ^(range: 3.0 to 7.0 kg/m^2^) and mean percent change in body weight of -12.0% (p = 0.006). Individual results are provided in Table 1.

**Table 1:** Effect of Diet on Individual Weight and Serum Metabolic Parameters

ID	Week	Weight	TChol	Trig	HDL-C	LDL-C	Glucose	HgbA1c	Insulin	Test	Free Test	Pct Free Test	LH/FSH	Became Pregnant
		lbs	mg/dl	mg/dl	mg/dl	mg/dl	mg/dl	%	μIU/ml	ng/dl	ng/dl	%		
1	1	267	229	99	62	147	89	5	11	41	0.61	1.49	1.7	
	10	242	196	83	50	129	83	5.2	6.3	43	0.67	1.57	0.4	
	24	226	237	87	57	162	69	5.0	4.5	47	0.57	0.57	0.9	
														
2	1	228	195	131	39	129	83	4.7	7.2	61	0.78	1.29	0.9	
	10	215	178	87	43	117	92	4.9	9.8	47	0.45	0.97	0.7	
	24	219	198	93	47	132	79	4.9	5.3	52	0.65	1.25	0.7	
														
3	1	168	183	95	66	98	82	5.2	10.9	41	0.84	2.06	3.3	
	10	155	234	59	50	172	87	5.3	5.3	38	0.66	1.76	2.5	*
	24	151	199	44	63	127	82	4.9	5.6	58	0.66	1.15	1.3	
														
4	1	277	231	108	48	161	131	8.6	72.7	85	3.23	3.8	2.0	
	10	252	193	75	40	138	86	6.8	24.2	21	0.62	2.97	0.4	
	24	237	190	54	41	138	75	6.5	19.5	41	0.77	2.97	1.0	
														
5	1	177	117	76	36	65	102	6.3	15.7	31	0.71	2.31	2.5	
	10	158	147	115	36	88	97	5.5	9.4	34	0.60	1.79	3.3	*
	24	148	153	88	35	100	91	5.8	6.1	42	0.78	1.88	2.5	

TChol = total cholesterol, Trig = triglycerides, HDL-C = high density lipoprotein cholesterol, LDL-C = low density lipoprotein cholesterol, HgbA1c = hemoglobin A1c, Test = testosterone, Free Test = free testosterone, Pct Free Test = percent free testosterone, LH = luetinizing hormone, FSH = follicle stimulating hormone

### Metabolic/endocrine parameters

From baseline to week 24, there were statistically significant reductions in percent free testosterone (from 2.19 to 1.70), LH/FSH ratio (from 2.23 to 1.21), and fasting serum insulin (from 23.5 to 8.2). The mean percent change in percent free testosterone was -30.0% (p = 0.04), in LH/FSH ratio was -36.0% (p = 0.03), and in insulin was -53.7% (p = 0.002). A reduction in serum insulin while maintaining fasting serum glucose (p = 0.10) and HgbA1c (p = 0.24) suggests an overall improvement in insulin resistance. Two women became pregnant during the study despite previous infertility problems.

Changes in serum lipid levels were also observed from baseline to 24 weeks, but none reached statistical significance. The mean percent change in triglycerides was -25.8% (p = 0.11), in HDL was -1.9% (p = 0.77), in LDL was +1.6% (p = 0.10), and in total cholesterol was +5.4% (p = 0.53).

### Blood pressure

During the 24 week period, the mean systolic blood pressure decreased 6.3 mm Hg (range: -2.5 to -15 mm Hg) and mean diastolic blood pressure decreased 9.6 mm Hg (range: -2.5 to -22.5 mm Hg) from baseline.

### PCOS-specific questionnaire

**Figure 1:**
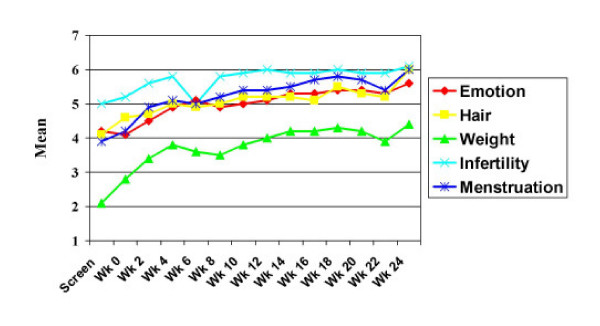
**Effect of Diet on PCOS-Q Scores. **The effect of a low-carbohydrate, ketogenic diet program on the mean polycystic ovary syndrome specific questionnaire (PCOS-Q) domain scores is shown over a 24 week period

The domain scores for the study duration are shown in Figure 1. There was a trend for a statistically significant improvement in domains of "hair," "infertility," and "menstruation" (p = 0.06 for all three domains, Wilcoxon signed-ranks test).

## Discussion

This pilot study showed that adherence to a low-carbohydrate, ketogenic diet led to improvement in body weight, percent free testosterone, LH/FSH ratio, fasting serum insulin, and symptoms in women diagnosed with PCOS over a six-month period. Further research is needed to determine if the benefits were from weight loss or from carbohydrate restriction specifically.

Our findings are similar to a previous clinical series of the use of a low (100 gram/d) carbohydrate, high saturated fat diet in 15 women with PCOS [21]. In that study, there was a 14.3 percent reduction in body weight (p = 0.008) and a reduction in fasting serum insulin from 24.2 μIU/ml to 12.2 μIU/ml from baseline to 24 weeks (p < 0.005). In our study, there was a 12.1% reduction in body weight (p = 0.006), and a reduction in insulin from 23.5 μIU/ml to 8.2 μIU/ml (p = 0.002). Taken together, these two clinical series support that formal research be directed toward carbohydrate restriction and PCOS.

The hyperinsulinemia of PCOS appears to increase androgen secretion from the ovary as well as to decrease circulating sex hormone binding globulin (SHBG) [22]. Our study suggested that a LCKD may lead to a reversal of these processes. We speculate that reduction in hyperinsulinemia due to the LCKD would decrease stimulation of ovarian androgen production as well as increase SHBG levels, synergistically limiting the amounts of circulating free-androgens in the serum. In addition, the reduction in LH/FSH ratio exhibited in our study may be indicative of endocrine re-normalization resulting from the LCKD intervention, due to an improvement in insulin sensitivity.

This pilot study was intended to assess whether further research should be directed toward this intervention. We show that for those individuals who were able to comply with the program, the effects were quite dramatic. This magnitude of weight loss with the resolution of PCOS symptoms is a desirable effect in any intervention. Other comparative studies are needed to determine if the effects are due to weight loss or to the specific dietary approach. Another limitation is that the hormonal measures were not taken at specified points during the menstrual cycle. Because none of the women were amenorrheic, these tests may have been confounded by menstrual cycle changes.

The LCKD assessed in this study was designed to simulate the most restrictive periods of several lay-press lifestyle books. Because of the baseline medical evaluation and ongoing medical supervision provided in this study, we allowed individuals to continue the LCKD over most of the six-month period. This approach differs from many of the popular programs, which recommend increasing the carbohydrate level after the first few weeks. For some participants, this dietary change was too demanding.

In summary, in this pilot study, a LCKD led to significant reductions in weight, percent free testosterone, LH/FSH ratio, and fasting serum insulin in women with obesity and PCOS over a six-month period.

## Competing interests

This study was partially funded by a research grant from the Robert C. Atkins Foundation. Dr. Yancy is supported by a Veterans' Affairs Health Services Research Career Development Award (RCD 02-183-1).

## Authors' contributions

EW and WY designed the study. EW, WY and JM performed and verified the statistical analysis. All authors participated in the data collection and manuscript writing, and approved the final manuscript.
